# Prevalence and factors associated with undernutrition and anaemia among school children in Durbete Town, northwest Ethiopia

**DOI:** 10.1186/s13690-015-0084-x

**Published:** 2015-08-10

**Authors:** Tilahun Alelign, Abraham Degarege, Berhanu Erko

**Affiliations:** Department of Microbial, Cellular and Molecular Biology, College of Natural Sciences, Addis Ababa University, P.O. Box 1176, Addis Ababa, Ethiopia; Department of Biology, Debre Birhan University, Debre Birhan, Ethiopia; Aklilu Lemma Institute of Pathobiology, Addis Ababa University, P.O. Box 1176 Addis Ababa, Ethiopia; Department of Epidemiology, Robert Stempel College of Public Health and Social Work, Florida International University, Miami, FL USA

**Keywords:** Undernutrition, Anaemia, School children, Northwest Ethiopia

## Abstract

**Background:**

Information about risk factors of undernutrition and anaemia is useful to design appropriate strategies to control the health problems. In this study, the prevalence and factors associated with undernutrition and anaemia were assessed among school children in Abchikeli and Ayalew Mekonnen Elementary Schools, northwest Ethiopia, in February and March 2010.

**Methods:**

A cross-sectional study was carried out among 384 school children. Stool samples were examined using single Kato-Katz slide and nutritional status was determined using anthropometry technique. A pre-tested standardized questionnaire was used to gather information on the socio-demographic and the socio-economic status of the school children. Multivariate logistic regression analysis was used to quantify the association of intestinal helminth infection and socio-demographic and socio-economic factors with undernutrition and anaemia.

**Results:**

Out of 384 children examined, 32.3 % were undernourished (27.1 % underweight and 11.2 % stunted) and 10.7 % were anaemic. The odds of stunting were approximately seven times higher in children of ages 10 to 14 [Adjusted Odds Ratio (AOR) = 6.93, 95 % CI = 2.60, 18.46] and 2.5 times higher in males [AOR = 2.50, 95 % CI = 1.24, 5.07] than children of ages 5 to 9 and females, respectively. The odds of underweight was three times higher in children who did not wash their hands before eating compared to those who did wash their hands [AOR = 3.13, 95 % CI = 1.19, 8.17]. The chance of anaemia was nine times higher in children who were infected with hookworms compared to those who were not infected with any helminth species [AOR = 8.87, 95 % CI = 2.28, 34.58]. The odds of being undernourished and anemic were similar among children with different socio-economic status.

**Conclusions:**

Undernutrition and anaemia are public health problems of school-age children in Durbete Town. Health education and provision of additional food supplements would be important to reduce the problem of undernutrition among school-age children in the town. Deworming of children in the town would also have additional impact on reducing the level of anaemia.

## Background

Undernutrition and anaemia are common public health problems among people in the developing regions. These diseases are particularly prevalent in children. About 25.4 % of school age children in the world are anaemic [1]. Closer to 90 % of undernourished children under-five years of age in the globe live in developing regions [2] and 40 % of school age children living in developing regions are anaemic [1]. Undernutrition and anaemia affect physical and mental development and immunity, increasing susceptibility of cases to infection and death [3, 4]. Undernutrition is responsible for the death of one-third of children (7.6 million children) in the globe every year [5].

Poverty, illiteracy, poor diet practice, genetic abnormalities, digestive difficulties, absorption problems and intestinal helminth infection are major risk factors associated with anaemia and undernutrition in children [6–10]. Due to the low socio-economic and sanitation conditions prevailing in Ethiopia, undernutrition and anaemia are common problems of citizens (particularly children) of the country. The country remains one of the most undernourished populations in the world [11]. About 44, 29 and 10 % of children under five years of age in Ethiopia were stunted, underweight and wasted, respectively [12]. Although the major risk factors of undernutrition and anaemia have been previously reported in different regions, the specific causes may vary with localities and time. In addition, causes of anaemia and undernutrition in developing regions are heterogeneous and complex. Hence, current information about risk factors of the problems for each region is helpful to design integrated, timely and appropriate strategies to effectively control the diseases. In this study, the prevalence and factors associated with undernutrition and anaemia were assessed among school children in Abchikeli and Ayalew Mekonnen Elementary Schools, northwest Ethiopia, in February and March 2010.

## Methods

### Study area and population

The study was conducted among school children (age: 5 to 15 years) in Abchikeli and Ayalew Mekonen Elementary Schools located in Durbete Town, northwest Ethiopia, in February and March 2010. The town is divided into 2 *kebeles* (the smallest administrative units). A total of 17,497 people live in the area (7781 males and 9716 females). According to the health center files, worm infections are the second (next to malaria) causes for visiting health centers for communities living in the town. The altitude of the town is 1500 masl. The mean annual temperature and annual rain fall ranges from 9 to 25 °C and 1400 mm to 1594 mm, respectively. The dominant soil type of the study area is brown and black loamy soil. The majority of the population of the town are engaged in mixed agricultural activities. Using multistage random sampling, a total of 384 (247 from Abchikeli and 137 from Ayalew Mekonnen Elementary Schools) school children were proportionally selected based on the size of the students of age 5 to 15 in each school enrolled in the study.

### Socio-demographic and socio-economic

Information about age, sex, education status of parents, grade level of the children, house floor nature, presence of latrine, hand washing habit before eating, drinking water source, place of residence and religious status of children was collected using a questionnaire. A pre-tested structured questionnaire was constructed in English and translated into Amharic. Then, the children were interviewed in Amharic.

### Stool collection and examination

The school children were provided with small plastic sheets and clean wooden applicator stick and informed to bring about 5 mg stool sample. The samples were then processed using Kato-Katz method and examined microscopically for ova of intestinal helminths [13]. Egg counts for hookworm were made on the spot within 30 to 45 min of slide preparation, whereas the specimens were quantitatively examined for the eggs of *A. lumbricoides* and *T. trichiura* within one week of specimen collection at the laboratory of Aklilu Lemma Institute of Pathobiology, Addis Ababa University. Eggs counted for each species of helminth were multiplied by 24 to estimate the number of eggs in a gram of stool and infection intensity was determined accordingly [14].

### Nutritional status

Weight and height were measured using a digital portable weighing calibrated SECA scale. The school children were weighed wearing light clothes (school uniforms) without shoes. The calibrated SECA balance scale has intervals/ sensitivity of 0.1 kg and 0.1 cm precision. Weighing scale was calibrated to zero before taking every measurement. Classification of the nutritional status of the children was made according to the WHO Growth Standards. Children were considered undernourished when they were either stunted (Z of height for age less than −2) or underweight (Z of body mass index for age less than −2) [15].

### Haemoglobin level determination

Blood samples from each child were collected by finger pricking after rubbing the finger-tip with sterile cotton (immersed in alcohol), and pricking it with a sterile disposable lancet. A drop of blood was then allowed to enter the optical window of the microcuvette through capillary action. The microcuvett was placed into the cuvett holder of a hemocue spectrophotometer (Hemocue HB 201 analyzer) and the concentration of haemoglobin was quantitatively determined in g/dl. Anaemic status was then determined accordingly. The haemoglobin cut-off levels indicating anemia were below 11.5 g/dl for children in the 5–11 years age group and below 12.0 g/dl for children in the 12–14 years age group [16].

### Data analysis

The data were entered, cleaned and checked using Excel 2007 and analysis was performed using STATA (version 10, Stata Corporation, College Station, Texas, USA). Proportions were used to describe the prevalence of intestinal helminth infections, stunting, underweight and anaemia. Z-test of proportions was used to test the difference in the prevalence of stunting, underweight and undernutrition between different age and sex groups. Multivariate logistic regression analysis was used to measure the strength of association of socio-economicand socio-demographic factors and intestinal helminth infections with undernutrition and anaemia. Ninety five percent confidence interval (CI) was calculated for the odds ratio values. Values were considered significant when P < 0.05.

### Ethical considerations

Ethical clearance was obtained from the Ethical Clearance Committee of the Department of Microbial, Cellular and Molecular Biology, College of Natural Sciences, Addis Ababa University. Permission to conduct the study was also obtained from Debub Achefer District Health Offices, educational authorities and school principals. The samples were collected from children who gave their assent and whose parents/guardians signed the written informed consent. All children who had intestinal helminth infections were treated with an appropriate dose of 500 mg mebendazole.

## Results

A total of 403 children were contacted of whom 384 brought stool samples and responded to the questionnaire. Of a total of 384 study participants examined, 32.3 % were undernourished and 10.7 % were anaemic (Table 1). About 27.1 % of the children were underweight while 11.2 % were stunted. The prevalence of stunting was significantly higher in males (P = 0.01) and children of ages 10 to 15 years (P < 0.01) compared to females and children of ages 5 to 9 years (Table 2), respectively. The difference in the prevalence of anaemia was not significant between sexes (P = 0.64) and age groups (P = 0.53).

**Table 1:** Characteristics of children in Abchikeli and Ayalew Mekonnen elementary, northwest Ethiopia, February and March 2010

Variables	Categories	Frequency	Percent
Age in years	5-9	175	45.6
	10-14	209	54.4
Sex	Male	172	44.8
	Female	212	55.2
Helminth infection	Yes	211	54.9
	No	173	45.1
Nutrition status	Undernourished	124	67.7
	Normal	264	32.3
Anaemic status	Anaemic	41	10.7
	Not anaemic	343	89.3
Family	1 to 3	207	53.9
Size	4-6	123	32.0
	≥7	54	14.1
Fathers	Literate	182	47.4
education	Illiterate^a^	202	52.6
Mothers	Literate	143	37.2
education	Illiterate^a^	241	62.8
Drinking	Tap	131	34.1
water	Well	148	38.5
sources	River/spring	105	27.4
Hand	Yes	361	94.0
Washing habit before eating	No	23	6.0
Shoe	Yes	86	22.4
Wearing	No	298	77.6
Presence	Yes	151	39.2
of latrine	No	233	60.7
House	Cement	21	5.5
Floor	Earthen	263	94.5
Residence	Urban	137	35.7
	Rural	247	64.3
Grade	1 to 3	279	72.7
	3 to 6	105	27.3
Religion	Christian	366	95.3
	Muslim	18	4.7

Iliterate^a^= can not read and write

**Table 2:** Prevalence of undernutrition and anaemia among children in Abchikeli and Ayalew Mekonnen Elementary Schools stratified by age and sex, northwest Ethiopia, February and March 2010

Variables	Number examined	Percent stunted (HAZ < −2)	Percent underweight (BAZ < −2 or WAZ < −2)	Percent undernourished (WAZ < −2 or HAZ < −2 or BAZ < −2)	Percent anaemia
Age					
5-9	175	4.57	30.86	31.43	7.43
10-14	209	16.75	23.92	33.01	13.4
Total	384	11.2	27.1	32.3	10.7
P-value		0.00	0.13	0.74	0.53
Sex					
Females	212	7.6	69.81	33.02	11.32
Males	172	15.7	76.74	31.4	9.9
P-value		0.01	0.13	0.74	0.64

WAZ = Z-scores for Weight-for-age- calculated when age from 5 to 10 years

HAZ = Z-scores for Length/height-for-age- calculated when age from 5 to 15 years

BAZ = Z-scores for BMI-for-age- calculated when age from 5 to 15 years

### Intestinal helminth infection, undernutrition and anaemia

The odds of stunting was seven times higher in children of ages 10 to 14 [Adjusted Odds Ratio (AOR) = 6.93, 95 % CI = 2.60, 18.46] and 2.5 times higher in males [AOR = 2.50, 95 % CI = 1.24, 5.07] than children of ages 5 to 9 and females, respectively. The chance of anaemia was approximately nine times higher in children who were infected with hookworm compared to those who were not infected with helminths [AOR = 8.87, 95 % CI = 2.28, 34.58] (Table 3). However, the difference in the prevalence of underweight was not significant between males and females and children of ages 5 to 9 and 10 to 14 years. Similarly, the difference in the prevalence of underweight and stunting were not significant between children who were infected and those not infected with intestinal helminths.

**Table 3:** Association of intestinal helminth infection, sex and age with undernutrition and anaemia among children in in Abchikeli and Ayalew Mekonnen elementary schools, northwest Ethiopia, February and March 2010

		Undernutrition			Anaemia		
Variables	Categories	Yes	No	AOR^a^ [95 % CI]	Yes	No	AOR^a^ [95 % CI]
Helminth infection	Non-infected	55	118	-	3	170	-
	*A. lumbricoides*	13	13	2.16 [0.85, 5.53]	0	26	NA
	Hookworm	44	101	1.42 [0.80, 2.52]	23	122	8.87 [2.28, 34.58]
	*T. trichiura*	0	5	NA	0	5	NA
	Any	69	142	1.28 [0.79, 2.09]	38	173	10.65 [2.96,38.39]
	Single	57	119	1.41 [0.85, 2.33]	23	153	8.11 [2.20, 29.88]
	Double	10	23	0.53 [0.17, 1.65]	15	18	43.56 [8.42, 225.55]
	Triple	2	0	NA	0	2	NA
Age in years	5-9	55	120	-	13	162	-
	10-14	69	140	1.36 [0.76, 2.43]	28	181	0.61 [0.22, 1.71]
Sex	Male	54	118	-	17	155	-
	Female	70	142	1.17 [0.73, 1.86]	24	188	1.06 [0.49, 2.26]

NA: not applicable

AOR^a^ (adjusted odds ratio): adjusted for age, sex, helminth infection, education status of parents, grade level of the children, house floor nature, presence of latrine, hand washing habit before eating, drinking water source, place of residence and religious status: based on two (for anaemia and undernutrition) independent multivariable regression models

### Association of socio-economic and socio-demographic factors with undernutrition and anaemia

The odds of underweight was three times higher in children who did not wash their hands before eating compared to those who did wash their hands [AOR = 3.13, 95 % CI = 1.19, 8.17]. However, the odds of underweight and stunting were similar between children with different family size, family education status, drinking water source, shoe wearing habit, living house floor nature, place of residence, latrine usage habit and grade level. Similarly, the chance of developing anaemia was similar between children with different socio-economic status (Table 4).

**Table 4:** Association of socioe-conomic and socio-demographic factors factors with undernutrition and anaemia among children in Abchikeli and Ayalew Mekonnen Elementary Schools, northwest Ethiopia, February and March 2010

		Undernutrition			Anaemia		
Variables	Categories	Yes	No	AOR [95 % CI]^a^	Yes	No	AOR [95 % CI]^a^
Family	1 to 3	74	133	-	20	187	-
Size	4-6	33	90	0.78 [0.45, 1.35]	13	110	0.61 [0.25, 1.48]
	≥7	17	37	0.94 [0.44, 1.99]	8	146	0.93 [0.32, 2.72]
Fathers	Literate	66	116	-	17	165	-
education	Illiterate^b^	58	144	0.88 [0.39, 1.99]	24	178	0.90 [0.29, 2.75]
Mothers	Literate	55	88	-	11	132	-
education	Illiterate^b^	69	172	1.33 [0.55, 3.22]	30	211	0.87 [0.22, 345]
Drinking	Tap	55	76	-	5	126	-
water	Well	42	106	0.64 [0.21, 1.97]	26	122	0.91 [0.05, 15.33
sources	River/spring	27	78	0.27 [0.02, 3.85]	10	95	0.68 [0.02, 23.08]
Hand	Yes	113	248	-	33	328	-
washing±	No	11	12	2.62 [1.02, 6.72]	8	15	2.73 [0.90, 8.24]
Shoe	Yes	34	52	-	1	85	-
Wearing	No	90	208	1.09 [0.52, 2.29]	40	258	5.91 [0.41, 85.95]
Presence	Yes	62	89	-	8	143	-
of latrine	No	62	171	0.60 [0.22, 1.64]	33	200	0.30 [0.04, 2.07]
House	Cement	11	10	-	2	19	-
Floor	Earthen	113	250	1.79 [0.54, 5.96]	39	324	4.13 [0.09, 195.52]
Residence	Urban	57	80	-	6	131	-
	Rural	67	180	1.19 [0.33, 4.36]	63	212	0.11 [0.00, 3.03]
Grade	1 to 3	89	190	-	24	255	-
	4 to 6	35	70	1.57 [0.81, 3.07]	17	88	2.23 [0.82, 6.10]
Religion	Christian	117	249	-	39	327	-
	Muslim	7	11	0.40 [0.09, 1.82]	2	16	0.61 [0.02, 15.63]

AOR^a^ (adjusted odds ratio): adjusted for age, sex, helminth infection, education status of parents, grade level of the children, house floor nature, presence of latrine, hand washing habit before eating, drinking water source, place of residence and religious status: based on two (for anaemia and undernutrition) independent multivariable regression models

Illiterate^b^: Can not read and write

Hand washing±: hand washing habit before eating

## Discussion

A total of 384 children enrolled in Abchikele and Ayalew Mekonnen Elementary Schools were examined and 32.3 % of them were found undernourished (27.1 % were underweight and 11.2 % were stunted) and 10.7 % were anaemic. Male and older age children were at risk of being stunted and those who did not wash their hands before eating were at risk of being underweight. The chance of being anaemic was higher in children who were infected with hookworm compared to those who were not infected with helminths. However, the prevalence of undernutrition and anaemia were similar between children with different socio-economic status and those who were infected with intestinal helminth and those were not infected.

The prevalence of undernutrition (underweight and/or stunting) in the current study was similar to a recent report among school-age children in Addis Ababa (30.9 %) [17]. The prevalence of underweight found out in the current study is also comparable with the prevalence documented in other regions of Ethiopia [18, 19]. However, some studies documented a higher prevalence of stunting (19.6 to 29.3 %) [17–20] and undernutrition (underweight and/or stunting) (42 to 46.7 %) [17, 19, 20] among school-age children in different places of the country. These differences could be due to variations in the factors predisposing to underweight and stunting in the different regions of Ethiopia. Stunting is chronic and associated with long term factors; howevere, being underweight is due to current and acute or chronic inadequate nutrition.

The prevalence of anaemia among school-age children in the current study is mild. However, a relatively high prevalence of anaemia (27.6 to 37.6 %) was observed among school-age children in a different regions of Ethiopia [21–23]. The etiologic agents of anaemia are different and could vary from place to place [16]. This may contribute to the differences in the prevalence of anaemia among school-age children in different regions of the country.

Male children of ages 10 to 14 years were at higher risk of being stunted. Similar previous studies also reported a higher prevalence of stunting in males than in females [18, 24–27] and in children of ages 10 to 14 years compared to those of ages 5 to 9 years [28–30]. These sex and age biases in the prevalence of stunting in the area could be related to community specific cultures. In the region, male children are usually more mobile and undertake different playing activities (e.g. sport) that make them loss grater energy from their body. On the other hand, females are usually give more attention to their personal hygiene than males, are less mobile in their behavior and stay at home, have more access to different food staff. As a result, females will be less vulnerable to undernutrition compared to males. Particularly, the physical activities are frequently practiced by older age children. On the other hand, the habit of not washing hands before eating among children was associated with a greater prevalence of underweight rather than stunting. Unlike stunting, which shows cumulative effects starting from a past period, being underweight is acute, indicating current nutritional status of children. Not washing hands before eating may cause acute bacterial or parasitic infection that would lead to diarrhea or mal-absorption problems and acute undernutrition.

Hookworm infection was independently associated with an increased risk of anaemia, as previously reported by many studies [31, 32]. However, the number of children infected with *A. lumbrcoides* and *T. trichiura* alone were too small to make valid statistical tests regarding the impact of these infections on anaemia and undernutrition. On the other hand, Soil Transmitted Helminths infection in general was not associated with undernutrition. This is in contrast to some previous studies [32–34]. The intensity of the majority of infections in the current study was light. As a result, the infections may not have significant impact on the nutritional status of the children.

Although previous studies linked low education status of mothers/fathers, drinking unprotected water, defecating in open fields, living in rural area and not wearing of shoes with undernutrition [35–39] and anaemia [40–42], similar associations were not observed in the present study. Other studies also reported that the aforementioned socio-economic factors do not increase the risk of undernutrition or anaemia [43, 44]. Different communities have different lifestyles, cultures, religions and environmental conditions. As a result, risk factors of undernutrition and anaemia may not be similar in all areas.

The use of single Kato Katz slide was a limitation in the present study because it might have underestimated the prevalence and intensity of infection in the study population. In addition, the cross-sectional nature of the study design hampers making conclusive conclusions regarding the impact of the socio economic factors and intestinal helminth infection on anaemia and undernutrition. Further community based longitudinal studies would be helpful to evaluate whether Soil Transmitted Helminths infection and poor socioeconomic, low education, poor sanitation status could lead to undernutrition and anaemia in children and adult among the communities in Durbete Town, North west Ethiopia.

## Conclusions

Undernutrition and anaemia are public health problems of school age children in Durbete town. Being male, age between 10 and 14 years and not washing hands before eating increases the risk of undernutrition. Hookworm infection was associated with a higher risk of anaemia. Health education and provision of additional food supplements could be important to reduce the problem of undernutrition among school age children in the town. Deworming of school age children in the town would also have additional impact on reducing the prevalence of anaemia among school age children living in Durbete town.
